# Optimization of bus stop layout considering multiple factors including passenger flow direction

**DOI:** 10.1371/journal.pone.0313040

**Published:** 2024-11-11

**Authors:** Guangchun Li, Lei Nie, Feng Gao, Zhenhuan He

**Affiliations:** School of Traffic and Transportation, Beijing Jiaotong University, Beijing, China; Stellenbosch University, SOUTH AFRICA

## Abstract

Bus stop layout typically requires consideration of urban population distribution, traffic conditions, and passenger flow demand to establish an efficient foundation for the bus system’s operation. Based on the above key factors, this paper introduces a strategic method to optimize the bus stop layout from a macro perspective in order to save passengers’ travel time and improve the attractiveness of the bus system. This approach accounts for the matching degree between the Origin-Destination (OD) direction of passengers and their walking direction heading to bus stops. Initially, we take into account factors such as the population and area of traffic districts, and urban road conditions. Utilizing the hypernetwork multidimensional data clustering method along with GIS technology, we construct an alternative set of bus stops based on the hypernetwork framework. This set serves as a reference for the positioning of newly built and moved bus stops. Subsequently, we develop a two-stage model for bus stop layout decision-making. The first stage focuses on determining the bus stop layout at the traffic district level, taking into account multi-factors including the passenger flow matching degree. The second stage is designed to mitigate the negative impact of bus stop optimization on the overall service level of the urban bus system. A case study conducted in XT city demonstrates the effectiveness of our approach. Post-optimization, there is a 15.83% increase in the alignment between passenger flow direction and bus stop layout. Additionally, the average travel time for passengers is reduced by 7.55 minutes.

## 1. Introduction

As the economy flourishes, the urban population and the number of motor vehicles are on the rise, exacerbating traffic congestion and environmental pollution. In numerous non-first-tier cities lacking a developed rail transit system, the public transportation system has emerged as the backbone of urban mobility. Integral to the urban transportation network, it is pivotal for the city’s smooth functioning and the ease of residents’ commutes. The design and operation of a conventional urban bus system encompass key elements such as the stop layout, route planning, and timetable formulation. The bus stop layout is a foundational design that precedes route planning and timetable formulation, holding significant strategic importance. For the majority of urban bus systems, the legacy of outdated urban traffic planning has resulted in the bus stop layout, with the system’s accessibility and convenience requiring enhancement. Consequently, a holistic consideration of various pertinent factors is imperative to refine the bus stop layout for improved efficiency and user experience.

Setting bus stops in a scientifically sound and rational way necessitates a four-pronged approach. Firstly, regarding passenger demand, the passenger flow direction should be taken into account, which is a key factor in this study. Many cities have multiple bus stops in various directions at large passenger hubs such as stations or shopping centers. Navigation software selects the nearest bus stop based on the relative positions of our starting point or destination to these locations, avoiding detours and significantly reducing travel time. However, in theoretical research, there is a dearth of scholars who systematically consider the passenger flow direction. We believe that quantifying this factor in the bus stop layout could greatly enhance the matching degree between urban passenger demand and bus stop layout, thereby improving the urban bus system’s service level. Secondly, the bus stop layout should consider several important factors such as urban road conditions and the population and area of traffic districts. Bus stops should not be located on internal roads within traffic districts. Based on technical standards and policies, they should be placed near the connection points between urban roads and internal roads of the traffic district to minimize passengers’ walking time. The higher the grade of the urban roads, the greater the vehicle traffic capacity; the larger the population in a traffic district, the greater the passenger demand, and the more inclined the bus stop placement should be. Additionally, when the area of a traffic district is too extensive, and passengers must walk for a longer duration, more bus stops should be set, considering the public welfare attribute of the public transport system. Thirdly, the essence of bus stop layout optimization lies in matching the demand of passenger flow with the supply of bus stops, and the core is to determine the relationship between the cost of stop optimization and passenger demand, aiming to maximize passenger demand satisfaction while minimizing the cost of stop optimization. Lastly, when optimizing the existing urban bus system, the impact of stop optimization on the travel speed of the bus network must be considered. Since bus stop optimization primarily involves building new stops, the extent to which these new stops reduce the travel speed of the bus network should be quantified. A feedback mechanism should be designed into the optimization method to prevent the occurrence of negative optimization outcomes.

Considering the passenger flow direction, urban road conditions, the population and area of traffic districts, the cost of building new bus stops, and the impact of stop optimization on the travel speed of the bus network, is a crucial engineering practice requirement in optimizing the bus stop layout and a key task in enhancing the efficient operation of the urban bus system.

## 2. Related work

The bus stop layout problem is considered to be a strategically important issue in the planning stage of the bus system, which largely determines the service scope and service efficiency of the bus system, and the bus travel service becomes attractive when the travel time of the bus system is comparable to that of a private car [1]. Bus stop spacing is the primary criterion for the optimization study of bus stop layout, and a bus stop layout with unreasonable spacing will make the service quality of the bus system low [2]. When bus stops are set up too centrally in a part of the area, it will increase the unnecessary vehicle dwell time, resulting in further lengthening of passengers’ waiting time at the stops and travel time on the bus, while the bus stop layout is too dispersed, will make the walking time from the starting place to the boarding stop or the alighting stop to the destination too long. This shows the importance of bus stop spacing, and some scholars input bus stop spacing based on design criteria and realistic considerations as an important parameter [2–4], so as to maintain a balance between stop coverage and bus travel speed, and Devunuri et al. [5] explored the reasonable setting range of bus stop spacing in terms of the problem of bus stop consolidation. The coverage or accessibility of bus stops is also the focus of many scholars in the evaluation of bus stop layout rationality. Stop coverage, that is the buffer zone around a bus stop, is a measure of the proportion of the population or area that can be served by a bus stop. Murray et al. [6] proposed three basic coverage siting models, the location set coverage problem model, the maximal coverage siting problem model, and the possibility of increasing the availability of a facility through the provision of backup coverage by a lower level of facility. The accessibility of bus stops not only depends on the walking distance of passengers, but is also related to some traffic facility factors, such as intersections and pavements, etc., and the passenger flow of bus stops is negatively correlated with accessibility. Horner et al. [7] proposed a density-based accessibility calculation method based on Temporal Geographic Density Estimation (TGDE) in order to estimate the level of accessibility of public transport. Lantseva et al. [8] proposed a model for public transport accessibility that is highly generalizable due to the use of highly relevant open data source crawls. These two accessibility evaluation methods analyze the urban bus system based on a macro perspective, while some other methods describe pedestrian accessibility for each bus stop. Corazza et al. [9] proposed a multi-step and multi-indicator approach for assessing the accessibility of bus stops, where data from different domains are obtained for a comprehensive assessment through the steps of selecting indicators and expert questionnaires. Hasan et al. [10] constructed a GIS-based accessibility model for bus stops, and in addition to considering different walking times, the authors superimposed socio-economic variables such as age and income to make the model more adaptable.

The optimization of bus stop layout is essentially a matching between the demand side of passenger flow and the supply side of bus stops, and the key lies in how to accurately describe the demand of passenger flow in different areas and find out the locations of bus stops that maximize the potential demand of bus flow. Li et al. [11] used the data of residence-work location and the number of inhabitants-employees for the potential demand, and thus the proposed bus stop supply-demand coupling method, and the proposed bus stop supply-demand coupling method can effectively identify bus stops with significant supply-demand imbalances. Taplin et al. [12] constructed a bi-objective optimization model and solved it using a genetic algorithm to find the stops that maximize passenger demand in the set of potential bus stops, and at the same time to find the shortest route connecting these stops. Established studies have produced many results for the problem of optimizing the bus stop layout in different scenarios. For bus corridors or bus lines, in terms of modelling, Medina et al. [13] decide on stop locations based on continuous and multi-period approximations of passenger demand in bus corridors, Zheng et al. [14] determine the optimal stop spacing by taking into account the cost of passenger boarding and alighting time, Moccia et al. [15] further extend the model by adding parameters such as optimal stop spacing and congestion penalties, Bargegol et al [16] used a meta-heuristic approach for stop siting modelling, while Jin et al. [17] proposed a road segment node unit transfer model based on two lanes, algorithmically, Zhu et al. [18] devised an improved Sequential Quadratic Programming (SQP) technique solving the stop siting model oriented to minimize the total travel time of the inhabitants, Ghasedi et al. [19] used the Hammersley sampling method as well as Genetic Algorithm (GA) and Particle Swarm Optimization (PSO) algorithms to generate non-uniform populations and solve the model, Duan et al. [20] proposed an artificial intelligence algorithm based site optimization method to integrate and analyze bus GPS data and swipe card data to optimize the bus stop settings, Bhuiya et al. [21] proposed a hierarchical analysis-based bus stop selection technique, and Yuan et al. [22] proposed a two-population adaptive immunity algorithm to solve a customized bus stop siting model. For urban bus networks or public transport systems, Delmelle et al. [23] integrated a Spatial Interaction Coverage (SIC) bus stop optimization model through a simulated annealing algorithm within a GIS framework, Zamanian et al. [24] proposed a multistep heuristic approach based on the established network as well as some of the traffic factors and constraints, constructing a multi-objective planning model to make decisions on bus stop siting, Otto et al. [25] proposed a two-stage dynamic planning heuristic for the Maximum Coverage Location Problem (MCLP), Wang et al. [26] constructed a bus stop model that considers the attraction range of the metro based on the idea of constructing a metro-dominated and bus-supplemented integrated urban transit system, and designed the Non-dominated Sorting Genetic Algorithm II (NSGA-II) to solve it, and Wang et al. [27] evaluated the accessibility of the service area they constructed based on the Voronoi diagram thus constructing a bus stop alternative set and proposed a complete bus stop layout optimization model. In addition to the line and network level, some scholars have also studied the bus stop optimization problem under certain specific factors or conditions. Ceder et al. [28] constructed a bus stop optimization model considering uneven terrain, Liu studied the bus stop layout problem based on the conditions of coordinated control of arterial lines [29], and also studied the optimization scheme based on the conditions of new road construction or reconstruction by considering the ideal layout or the local fine-tuning [30].

In summary, there are many research results and rich research scenarios for the bus stop layout optimization problem, scholars have constructed two-layer, two-stage, or multi-objective optimization models based on their respective considerations, such as travel demand, transportation facilities, land use, population density, etc., with the objectives of optimal stop spacing, maximum stop coverage or minimum passenger travel time, and also designed different heuristic algorithms or intelligent algorithms are designed for the solution. However, most of the current models and algorithms are applied to a certain bus line or bus corridor in the city, while there are not many studies on the optimization of the overall bus stop layout in the city, and there is a lack of a complete optimization method for bus stop layout that integrates multiple factors. This study aims to propose a two-stage optimization model for bus stop layout and design an algorithm to solve the problem by using GIS technology and hypernetwork theory, taking into account the passenger flow direction, urban road conditions, the traffic district population, area, the cost of building bus stops, and the impact of the stop optimization on the travel speed of the existing bus network, etc. The bus stop layout optimization model we constructed focuses on improving the matching degree between passenger flow direction and bus stop layout. The model takes the basic road network, traffic district division, passenger flow data, and bus stop alternative set as key inputs to make decisions on building, removing, or moving bus stops, and provides the precise geographic coordinates of the newly built or moved stops, and the corrected inter-stop passenger flow matrices. The main innovations of this paper are as follows:

1. We construct an initial bus stop alternative set using GIS technology, quantify factors such as traffic district population, area, and urban road conditions of the stops using hypernetwork theory, and design an algorithm to merge the alternative set with the objectives of bus stop spacing and stop coverage from generating a more reasonable bus stop alternative set.
2. We construct a two-stage optimization model and design a solution algorithm for it, which, in the first stage improves the matching degree between the passenger flow direction and the bus stop layout in the traffic district, and in the second stage, reduces the impact of stop optimization on the overall service level of the urban bus system.

The rest of the paper is structured as follows: Section III describes the process of constructing the bus stop alternative set and the two-stage model and solution algorithm for bus stop layout optimization. Section IV conducts a case study based on actual data and analyzes the optimization results in terms of metrics. Section V summarizes the research results of this paper and discusses the shortcomings of the study.

## 3. Model and algorithms

### 3.1. Modeling methodology

At the outset, we briefly introduce our modeling methodology. We first construct an alternative set of bus stops based on hypernetwork multidimensional data clustering methods, considering factors such as regional population, area, and urban road conditions, for the model to decide on the building and moving of bus stops. Then, considering factors such as the matching degree between passenger flow direction and bus stop layout and the overall service level of the urban bus system, we construct a two-stage model for the bus stop layout, deciding on the building, moving, and removal of bus stops.

For clarity, we need to define some concepts used in this paper here. The source district refers to a continuous area such as a residential area, shopping mall, office building, etc., which has no urban roads and no bus lines running inside. The source district is a more precise area than the traffic district division, and generally, the source district is included in the traffic district. The connecting road refers to the road that connects the source district with the urban road for citizens to walk. The node refers to the intersection of the connecting road and the urban road, which we consider as the initial alternative set of bus stops, the set of these nodes is called the initial node set.

**Fig 1:**
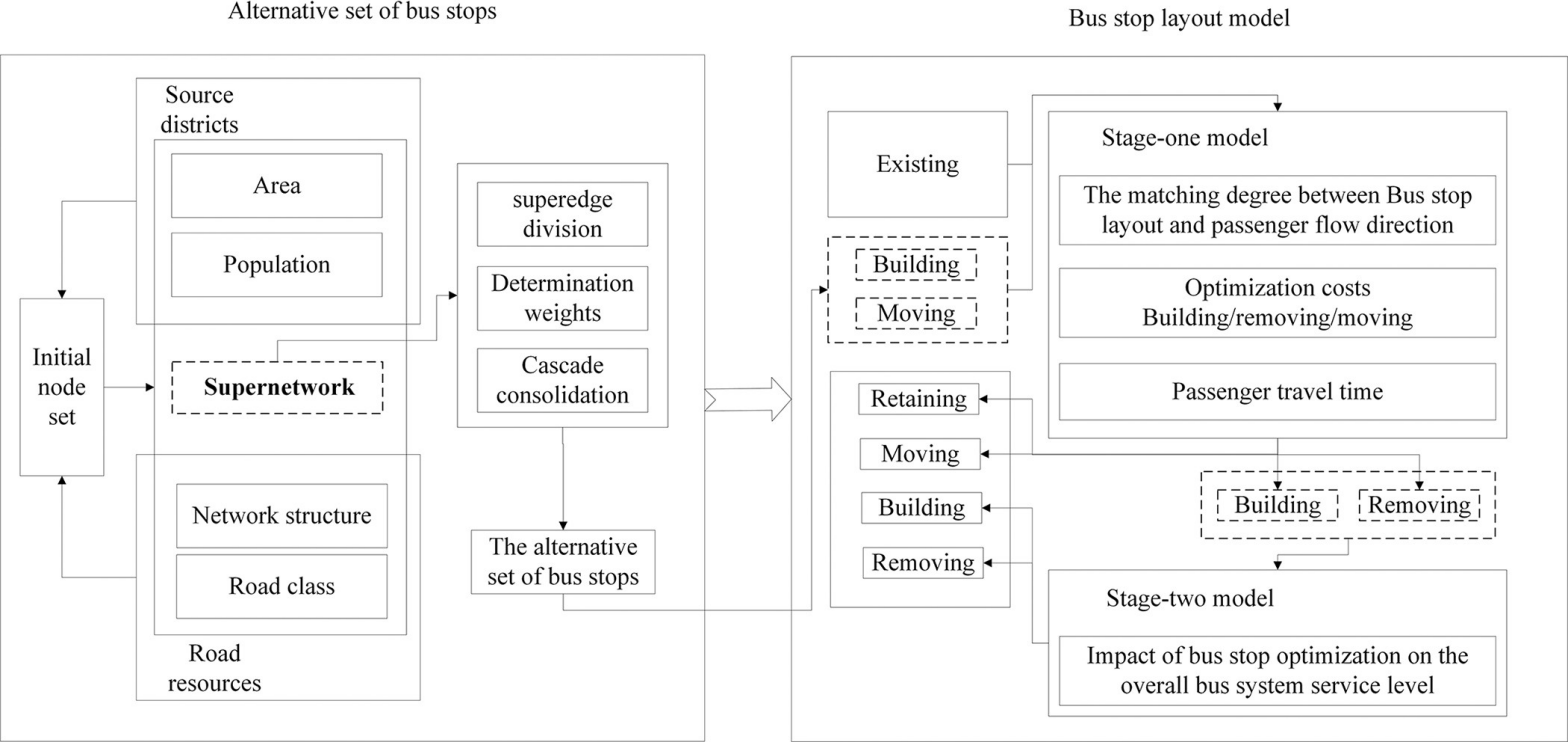
Optimization process of the bus stop layout.

Based on the above brief methodology overview and conceptual definitions, the bus stop layout optimization methodology in this paper mainly includes two steps: constructing an alternative set of bus stops and optimizing the bus stop layout. The optimization process of the bus stop layout is shown in Fig 1.

In the construction of the alternative set of bus stops, we first use GIS technology to construct the initial node set, and then use the population, area, and urban road conditions of the source district to construct the hypernetwork and divide the hyperedges. Next, to adapt to the effective coverage requirements of bus stops, we design an algorithm to merge the nodes of the hypernetwork level by level to generate an effective alternative set of bus stops.

In the construction of the bus stop layout model, we first consolidate the existing bus stop passenger flow OD data to the traffic district level, clarifying the direction and scale of the demand for bus travel. Then, we input the demand for bus travel and the alternative set of bus stops into the constructed two-stage model. The first stage optimizes at the traffic district level to improve the matching degree between the bus stop location in the traffic district and the passenger flow direction. The second stage optimizes at the bus system level with the newly built and removed stops decided by the first stage model as input, starting from the perspective of minimizing the impact of stop adjustments on its overall service level, and making the final decisions.

### 3.2. Model assumptions

The rational bus stop layout is the cornerstone of bus line planning, providing the necessary conditions for the efficient transportation of passenger flow between the main origins and destinations in the city. The optimization of bus stop layout should not only serve the planning of bus lines but also transcend the limitations of existing bus lines to achieve a higher level of traffic system optimization. Based on the previous description of the bus stop layout optimization problem, to highlight the focus of the research and ignore unimportant influencing factors, we need to make some relevant assumptions:

1. When planning bus lines, bus companies usually set a few different types of bus stops according to different operating areas or models. We consider all bus stops as one category, that is, regular bus stops.
2. When operating bus lines, bus companies usually set two identical bus stops on both sides of the road to achieve bidirectional operation of buses. When optimizing the bus stop layout, we consider such a pair of bus stops as one, that is, we do not consider the up and down directions.
3. When traveling by bus, residents of the traffic district tend to choose bus stops that are in the same direction as their travel and have a shorter walking time.
4. The construction of newly built bus stops may attract passenger flow from surrounding stops. When the passenger flow diverts from the existing bus stops to the newly built bus stops can match the walking direction to the stops with the OD travel direction, it is considered that this part of the passenger flow will divert between stops. No quantitative study will be conducted for the time being on the increase in passenger flow due to the ease of travel resulting from the optimization of the bus stops.

### 3.3. Construct bus stop alternative set

In the context of optimizing the bus stop layout, traditional methods utilize graph theory, considering each bus stop as a node and edges as relationships between pairs of nodes. These methods can only represent the characteristics of each node by aggregating information from neighboring nodes. Hypernetwork, an extension of the concept of nodes and edges, allows connections between any number of nodes. They can capture multi-dimensional, higher-order associations between nodes, thus expressing more complex relationships. In this section, the purpose of constructing an alternative set of bus stops is to provide choices for the model’s decision-making regarding the newly built and removed stops. hypernetwork has good applications in multifactor clustering, which can aggregate various factors of bus stop layout to output reasonable alternative bus stops.

**Fig 2:**
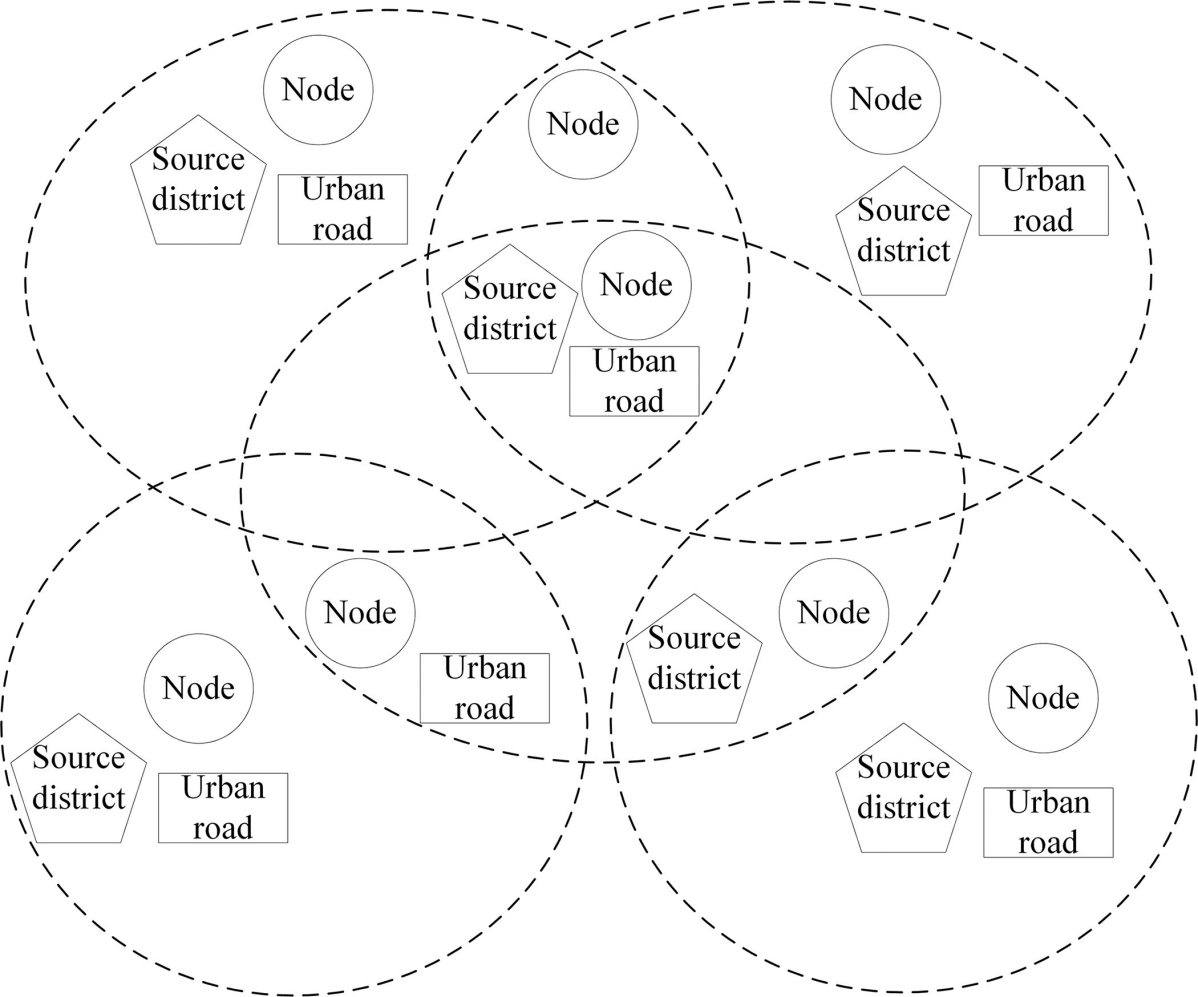
Node set hypernetwork.

Hypernetwork, also known as the hypergraph, is a generalization of the graph. Let the hypernetwork be *H* = (*V*,*E*,*W*), containing three sets, where *v* is the set of nodes with *V* = {*v*_1_,*v*_2_,…,*v*_*n*_}, *E* is the set of hyperedges with*E* = {*E*_1_,*E*_2_,…,*E*_*m*_}, *W* is the set of weights with *W* = {*w*(*E*_1_),*w*(*E*_2_),…,*W*(*E*_*m*_)}, and each hyperedge is assigned a weight. We use the source district population, area, and surrounding urban road conditions as the basis for clustering, and perform hypernetwork clustering on the initial node set, and the relationship between the nodes, the source districts, and the urban road is expressed using a hypernetwork as shown in Fig 2.

If the source district population is large or the district area is large, more bus stops should be provided around the district to meet passenger travel demand and reduce the walking time to the stops. The population of the source districts can be divided into *P*^1^ (greater than the district population threshold) and *P*^2^ (less than the district population threshold) according to the population threshold, and *U*^1^ (greater than the district area threshold) and *U*^2^ (less than the district area threshold) according to the area threshold. The critical values and critical coefficients of the population and area of the source districts can be determined using Pareto analysis, and we introduce the determination method using population as an example. The source districts are sorted according to population from largest to smallest, starting from the first source district, the first *n* source cells are summed up, and when the sum of their populations is greater than 75% of the total population of the study area, the population value corresponding to the *n*th source district is selected as the critical value of population, and the ratio of the population value of the *n*th source district to the first source district is used as the critical coefficient. Set the population and area criticality coefficients for the source district as *α* and *β*.

Priority should also be given to the provision of bus stops on urban roads of higher grades because of their greater capacity and higher passenger travel demand. Urban road grade is generally divided into five levels: expressway, trunk road, secondary road, tertiary road, and other roads, which is to express sequentially as *e*^1^,*e*^2^,*e*^3^,*e*^4^,*e*^5^.

There are 20 possible groups of orthogonality, in terms of the source district population scale, area scale, and urban road class. Combined with the characteristics of hypernetwork clustering, we divide the hypernetwork composed of nodes, source districts, and urban roads into 5 subsets, and each subset is the hyperedge of the hypernetwork, which is divided as in Table 1. We define the hyperedge level with *H*_1_>*H*_2_>*H*_3_>*H*_4_>*H*_5_.

**Table 1:** Division of hyperedges.

Node classification	Hyperedge division	Node classification	Hyperedge division
*G*(*P*^1^,*U*^1^,*e*^1^)	*H*_1_	*G*(*P*^1^,*U*^2^,*e*^3^)	*H*_3_
*G*(*P*^2^,*U*^1^,*e*^1^)	*H*_1_	*G*(*P*^2^,*U*^2^,*e*^3^)	*H*_4_
*G*(*P*^1^,*U*^2^,*e*^1^)	*H*_1_	*G*(*P*^1^,*U*^1^,*e*^4^)	*H*_3_
*G*(*P*^2^,*U*^2^,*e*^1^)	*H*_2_	*G*(*P*^2^,*U*^1^,*e*^4^)	*H*_4_
*G*(*P*^1^,*U*^1^,*e*^2^)	*H*_1_	*G*(*P*^1^,*U*^2^,*e*^4^)	*H*_4_
*G*(*P*^2^,*U*^1^,*e*^2^)	*H*_2_	*G*(*P*^2^,*U*^2^,*e*^4^)	*H*_5_
*G*(*P*^1^,*U*^2^,*e*^2^)	*H*_2_	*G*(*P*^1^,*U*^1^,*e*^5^)	*H*_4_
*G*(*P*^2^,*U*^2^,*e*^2^)	*H*_3_	*G*(*P*^2^,*U*^1^,*e*^5^)	*H*_5_
*G*(*P*^1^,*U*^1^,*e*^3^)	*H*_2_	*G*(*P*^1^,*U*^2^,*e*^5^)	*H*_5_
*G*(*P*^2^,*U*^1^,*e*^3^)	*H*_3_	*G*(*P*^2^,*U*^2^,*e*^5^)	*H*_5_

After dividing the hyperedges of the hypernetwork, according to the definition of the weighted hypernetwork, we need to assign a weight to each hyperedge of the hypernetwork. The weight of a node in the hypernetwork with {*H*_1_,*H*_2_,*H*_3_,*H*_4_,*H*_5_} is {*μ*_1_,*μ*_2_,*μ*_3_,*μ*_4_,*μ*_5_}, which indicates the degree of importance of the node; considering the interrelationship between different classes of population (area) of the source districts, it is defined as *μ*(*U*_1_) = (1+*α*)/2,*μ*(*U*_2_) = *α*/2, *μ*(*P*_1_) = (1+*β*)/2, and *μ*(*P*_2_) =*β*/2; taking into account the capacity of different classes of roads and the role of the bus system, it is defined as *μ*(*e*_1_) = *μ*(*e*_2_) = 1, *μ*(*e*_3_) = 0.8, *μ*(*e*_4_) = 0.7, and *μ*(*e*_5_) = 0.6. The weight of the hyperedge is to take the weighted average of the weight values of all groups in the hyperedge, as shown in Table 2.

**Table 2:** Hyperedge weights.

*H*_*n*_	*μ*_*n*_	Weight
*H*_1_	*μ*_1_	αβ+0.75α+0.75β+0.5
*H*_2_	*μ*_2_	0.95αβ+0.45α+0.45β+0.2
*H*_3_	*μ*_3_	0.825αβ+0.375α+0.375β+0.175
*H*_4_	*μ*_4_	0.7αβ+0.325α+0.325β+0.15
*H*_5_	*μ*_5_	0.625αβ+0.15α+0.15β

The last thing we need to do is to merge the nodes of the hypernetwork level by level, performed between each level of nodes and the nodes whose level is greater than or equal to it. The merge is based on the minimum station spacing of the road network, if the shortest distance of the road network between two nodes is less than it, then they need to be merged according to certain steps. For example, suppose node *i* and node *j* need to be merged, the shortest distance of the road network between them is *l*, their weights are *μ*_*i*_ and *μ*_*j*_, and their hyperedge are *H*_*i*_ and *H*_*j*_. Then, the merged node *k* is located at a position along the shortest path of the road network between nodes *i* and *j*, moved by a distance *l μ*_*j*_(*μ*_*i*_+*μ*_*j*_) from node *i*. Its weight is *μ*_*i*_+*μ*_*j*_, and its hyperedge is *max*{*H*_*i*_,*H*_*j*_}.

### 3.4. Two-stage optimization model

**Fig 3:**
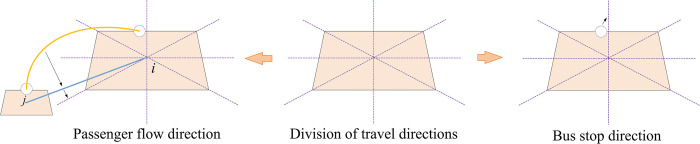
Division of travel directions in traffic districts.

Before constructing the bus stop layout optimization model, we need to pre-process the passenger flow data and merge the passenger flow of the same traffic district and travel direction. We take the center of the traffic district as the origin and divide the traffic district into eight travel directions, as shown in Fig 3. Based on the affiliation between bus stops and traffic districts, we first categorize bus stops into the corresponding direction of the traffic district they belong to. Then, we consolidate the passenger flow OD between bus stops into the passenger flow OD between traffic districts and divide it into the travel direction corresponding to the generating and attracting districts based on their relative positions. For example, the passenger flow from district *i* to district *j* with the volume of *q*_*ij*_, which is in the southwest direction relative to district *i*, is thus divided into the southwest-bound passenger flow of district *i* and the northeast-bound passenger flow of district *j*. It can be observed that no bus stop is set in the southwest direction of district *i*, causing a detour for this part of the passenger flow.

We define the sets, parameters, objective functions, and decision variables used in this paper, as shown in Table 3.

**Table 3:** Symbols definition.

Symbols	Meanings
o**Sets**	
*G*	The set of all traffic districts, *g*∈*G*
*D*	The set of all travel directions, *d*∈*D*
*M*	The set of all bus stops, *m*∈*M*
*M*_*g*_	The set of all bus stops in district *g*, *mg*∈*M*_*g*_
$T_{g}^{mg}$	The set of walking time to alternative bus stops of stop *mg* in district *g*.
**Parameters**	
$q_{im}^{d}$	OD passenger flow from stop *i* to *m*, with travel direction *d* (relative to the traffic district where stop *i* is located).
$v_{g}^{d}$	Whether there is a bus stop in travel direction *d* of district *g*, 1 if yes, 0 otherwise.
*w*_*ope*_	Operating cost of a bus stop.
*w*_*blt*_	Building cost for a new bus stop.
*q*_*up*_	The upper limit of the demand for inconvenient passengers.
*q*_*down*_	The lower limit of the demand for passengers at retained stops.
*d*_*max*_	The upper limit on the number of newly built bus stops in district *g*.
*d*_*min*_	The lower limit on the number of bus stops in district g.
*γ*	The maximum proportion of bus stops that can be removed in district *g*.
*q*_*a*_,*q*_*r*_	Amount of decrease or increase in inconvenient passenger flow resulting from the building or removal of bus stops
*t*_*a*_,*t*_*r*_	Amount of increase or decrease in travel time on bus routes resulting from the building or removal of bus stops
*q*_min_	The lower limit of net reduction of inconvenient passenger flow
*t*_max_	The upper limit of net increase in traveling time on bus lines
*f*_*a*,max_,*f*_*r*.max_	The upper limit on the number of bus stops to be built or removed
**Objectives**	
*Z*_1_,*Z*_2_,*Z*_3_	The objective for building, moving, and removing bus stops.
*Z*_4_	The objective for the second stage.
**Variables**	
$f_{g}^{d}$	Whether a new bus stop needs to be built in travel direction *d* of district *g*, 1 if needed, 0 otherwise.
$d_{g}^{mg}$	Whether stop *mg* in district *g* needs to be removed, 1 if needed, 0 otherwise.
$L_{g}^{mg}$	Whether the alternative stops *mhg* of stop *mg* in district *g* is selected, 1 if selected, 0 otherwise.
*f*_*a*_,*f*_*r*_	The final decision of building and removing bus stops from the decision-making of the first stage. 1 if determined, 0 otherwise.

#### 3.4.1. Stage-one model: Bus stop layout considering multi-factors focusing on the passenger flow matching degree

In the first stage of the optimization model, we consider the matching degree between the bus stop layout in the traffic district and the passenger flow direction. Passenger flow in the traffic district is divided into eight travel directions. The bus stop layout should be as consistent as possible with the walking direction of passengers within the traffic district, while also considering the connectivity with the internal roads of the traffic district to reduce passengers’ walking time. If there is a large passenger flow demand in a certain travel direction in a traffic district without a stop, it is necessary to consider building a bus stop in that direction. If the walking time to a certain bus stop in a traffic district is too long, it is necessary to consider moving the bus stop to a better location. The bus stop layout should also consider the necessity of bus stops based on passenger demand. If the passenger demand at a certain bus stop is less than the set threshold, it is necessary to consider removing the stop. Based on the alternative set of bus stops, the model for building, removing, and moving bus stops is constructed respectively in this stage, optimizing the selection of newly built and moved stops in the alternative set. Finally, the optimization results of the newly built and removed bus stops are used as the input for the second stage model.

The objectives include:

$$min\;Z_{1}=\sum_{d\in D}w_{blt}\cdot f_{g}^{d},g\in G$$ (1)

$$min\;Z_{2}=\sum_{mg\in M_{g}}w_{ope}\cdot (1-d_{g}^{mg}),g\in G$$ (2)

$$min\;Z_{3}=\sum_{mg\in M_{g}}L_{g}^{mg}\cdot T_{g}^{mg},g\in G$$ (3)

Eq 1 is the objective of the building bus stop model, which represents the sum of the costs of building bus stops in the traffic district, Eq 2 is the objective of the removing bus stop model, which represents the sum of the costs of removing bus stops in the traffic district, and Eq 3 is the objective of the bus moving stop model, which represents the sum of the walking arrival times at the existing bus stops in the traffic district.

Constraints include:

1. Constraints for building new bus stops
$$(1-f_{g}^{d})\cdot (1-v_{g}^{d})\cdot \sum_{m\in M}\left(\sum_{i\in M_{g}}q_{im}^{d}+\sum_{j\in M_{g}}q_{mj}^{d}\right)\leq q_{up},g\in G,d\in D$$ (4)
$$f_{g}^{d}+v_{g}^{d}\leq 1,g\in G,d\in D$$ (5)
$$\sum_{d\in D}f_{g}^{d}\leq d_{max},g\in G$$ (6)
2. Constraints for removing bus stops
$$\sum_{d\in D}\left(\sum_{i=mg,m\in M}q_{im}^{d}+\sum_{j=mg,m\in M}q_{mj}^{d}\right)\geq (1-d_{g}^{mg})\cdot q_{down},g\in G,mg\in M_{g}$$ (7)
$$\sum_{mg\in M_{g}}\left(1-d_{g}^{mg}\right)\leq \gamma \cdot \sum_{mg\in M_{g}}1,g\in G$$ (8)
$$\sum_{mg\in M_{g}}1-d_{g}^{mg}\geq d_{min},g\in G$$ (9)
3. Constraints for moving bus stops
$$T_{g}^{mg}\times I=1$$ (10)
  Eq 4 indicates that the number of inconvenient passengers in a travel direction of a traffic district should not be greater than the upper limit value, otherwise, new bus stops should be built in that travel direction, Eq 5 prohibits building stops in the travel direction of the traffic district where bus stops have already been set up, Eq 6 restricts the number of newly built bus stops in the traffic district, Eq 7 indicates that the existing bus stops should reach a set lower threshold of passenger flow, otherwise their removal should be considered, Eq 8 limits the proportion of removed bus stops in the traffic district, Eq 9 indicates that removal of a stop cannot make the number of bus stops in the traffic district less than the lower limit value, and Eq 10 indicates that only one of each existing bus stop and its corresponding alternative bus stops can be selected.

#### 3.4.2. Stage-two model: Decision on the bus stop layout considering the bus service level

In the second stage of the optimization model, we consider the impact of all newly built and removed bus stops in the traffic districts on the passenger service and the travel speed of the overall bus network. It is assumed that all existing bus lines passing through the newly built bus stops need to stop here. Therefore, while newly built bus stops provide services for inconvenient passengers, they will also increase the travel time of bus lines. Conversely, although removing bus stops will increase the number of inconvenient passengers, it will also reduce the travel time of some bus lines. Based on the optimization results of the first stage model, this stage calculates the amount of inconvenience passenger flow reduced(increased) by each newly built (removed) bus stop and the travel time of the bus line increased (reduced), and then input optimization model, deciding the final newly built and removed bus stops.

The objective is:

$$max\;Z_{4}=\frac{\sum_{a\in A}q_{a}\cdot f_{a}-\sum_{r\in R}q_{r}\cdot f_{r}}{\sum_{a\in A}t_{a}\cdot f_{a}-\sum_{r\in R}t_{r}\cdot f_{r}}$$ (11)

Eq 11 represents the ratio of the net reduction of inconvenient passenger flow and the net increase in bus line travel time brought by building and removing bus stops.

Constraints include:

$$q_{\min}<\sum_{a\in A}q_{a}\cdot f_{a}-\sum_{r\in R}q_{r}\cdot f_{r}$$ (12)

$$\sum_{a\in A}t_{a}\cdot f_{a}-\sum_{r\in R}t_{r}\cdot f_{r}<t_{\max}$$ (13)

$$f_{a}<f_{a,max}$$ (14)

$$f_{r}<f_{r,max}$$ (15)

Eq 12 indicates that the net reduction of inconvenient passenger flow should not be less than the lower limit value, and Eq 13 indicates the net increase in bus line travel time should not exceed the upper limit value. Eq 14 and Eq 15 restrict the maximum number of newly built and removed bus stops, respectively.

### 3.5. Solution algorithms

We designed two algorithms, the generation of the bus stop alternative set and the solution for the two-stage optimization model of bus stop layout.

The generation algorithm for the bus stop alternative set includes four key steps:

Step 1: Initial the node set based on the connecting roads of source districts and the urban roads;
Step 2: Based on the multi-factors of bus stop layout optimization, using the multi-factor clustering principle of hypernetwork, construct the hypernetwork and divide the hyperedges;
Step 3: Determine the hyperedge weights and classify the alternative stops according to the road grade, and the population and area of the source district;
Step 4: Optimize the classified stop set by a merging method to generate the bus stop alternative set.

The generation algorithm of the bus stop alternative set is described in detail as follows:

***Algorithm 1*: *The generation algorithm of the bus stop alternative set***

***Input***: *Road network*, *source districts*

***Output*:** *The alternative set of bus stops*

***Step1 Node set initialization***

*Based on the intersection points of the connecting roads of source districts and the urban roads*

*generate an initial node set*, *expressed in m*∈*M*.

***Step2 Hypernetwork construction and hyperedge division***

*Step 2*.*1 Sort the source districts by population (area) from largest to smallest*.

*Step 2*.*2 Select the value corresponding to the population (area) sum reaching 75% as the*

*population (area) threshold value P*_*b*_(*U*_*b*_) *and critical coefficient α*(*β*).

*Step 2*.*3 Construct the hypernetwork with nodes*, *source districts*, *and connecting roads*. *Divide*

*the hypernetwork into hyperedges h*∈*H*, *based on road level*, *source district population and*

*area threshold values to obtain the alternative set mh*∈*M*_*h*_.

***Step3 Hyperedge weight determination***

*Calculate the weights of the road level*, *source district population*, *and area*, *which are*

*respectively expressed in μ*(*e*_*i*_)、μ(*P*_*i*_)*、μ*(*U*_*i*_).

***for*** *h* ***in*** *H*

*Calculate the weighted average value of the group weight numbers at the h level*, *obtain the*

*h-level hyperedge weight μ*_*i*_, *and set it as the initial weight for each level hyperedge node*.

***Step4 Hierarchical merging of hypernetwork nodes***

Set mh*∈*M*_h_*as the set of nodes in the hyperedges with a level greater than or equal to h.

***for*** *h* ***in*** *H*

***for*** *mh* ***in*** *M*_*h*_

 ***for*** mh* ***in*** *M*_h*_

 Calculate the shortest distance between nodes mh and mh*.

 ***if*** *the shortest distance does not meet the minimum station spacing*

*Merge the two nodes*, *and calculate the location and weight of the merged node*.

The solution algorithm for the two-stage optimization model of bus stop layout includes five key steps:

Step 1: Calculate to which traffic districts the bus stops belong and their directions in the districts;

Step 2: Consolidate bus stop passenger flow into traffic district passenger flow based on boarding and alighting stops.

Step 3: Based on the matching degree of passenger flow direction, optimize the bus stop layout by newly building, removing, or moving bus stops around the traffic districts;

Step 4: Overall optimization of the bus stop layout based on the travel speed impact of the bus network;

Step 5: Correct the passenger flow matrix between bus stops according to the inter-stop diversion of passenger flow.

The solution algorithm for the two-stage optimization model of bus stop layout is described in detail below:

***Algorithm 2*: *The solution algorithm for the two-stage optimization model*.**

***Input*:** *Road network*, *traffic districts g*∈*G*, *existing bus stops m*∈*M*, *alternative set of bus*

*Stops mh*∈*M*_*h*_, *and bus stop passenger flow q*∈*Q*.

***Output*:** *bus stop set and bus stop passenger flow*.

***Step1 Bus stop direction division***

*Assume the existing bus stops in traffic district g are mg*∈*M*_*g*_.

***for*** *m* ***in*** *M*

 *Identify traffic district g which bus stop m belongs to*.

 *M*_*g*_ = *M*_*g*_+{*m*}

 *Calculate the direction of bus stop m in the traffic district g*.

***Step2 Consolidation of passenger flow***

***for*** *q* ***in*** *Q*

*Consolidate bus stop passenger flow q into traffic district passenger flow based on*

*boarding and alighting stops*.

*Determine the travel direction of q in the traffic district*.

***Step3 Optimization of traffic district bus stop layout model***

***for*** *g* ***in*** *G*

*Let the alternative set of bus stops in the traffic district g be* $mhg\in M_{h}^{g}$, *and the new*

*alternative set of newly built and removed bus stops generated by the first stage model be*

mh*∈*M*_h*_.

***for*** *mh* ***in*** *M*_*h*_

 ***if*** *the alternative bus stop mh is located in the traffic district g*

$M_{h}^{g}=M_{h}^{g}+\{mh\}$

 *Calculate the direction of mh in the traffic district g*.

 ***if*** *there is an existing bus stop mg in the direction of mh*.

 *Calculate the walking arrival time to the bus stop mh*.

*Input the first stage model to decide whether to build*, *remove*, *and move the bus stops*.

***if*** *a new bus stop is required*

$M_{h}^{g}=M_{h}^{g}\backslash \{mhg\}$

*M*_h*_ = *M*_h*_+{*mhg*}

 ***for*** *mg* ***in*** *M*_*g*_

***if*** *bus stop mg needs to be removed*

 *M*_h*_ = *M*_h*_+{*mg*}

***if*** *bus stop mg needs to be moved*

$M_{h}^{g}=M_{h}^{g}\backslash \{mhg\}$

*M* = *M*\{*mg*}

 *Move mg to mhg*.

***Step4 Overall optimization of the bus stop layout model***

***for*** mh* ***in*** *M*_h*_

 ***if*** mh* is the newly built bus stop

 *Calculate the reduction of inconvenient passenger flow and the increase in travel time on*

 bus lines resulting from mh*.

 ***if*** mh* is the removed stop

 *Calculate the increase in inconvenient passenger flow and the reduction in travel time on*

 bus lines resulting from mh*.

*Input into the second stage model to decide whether bus stops in the new alternative*

*set M*_h*_should eventually be built or removed.

***for*** mh* ***in*** *M*_h*_

 ***if*** mh* is the finalized bus stop to be built.

 *M* = *M*+{mh*}

 ***if*** mh* is the finalized bus stop to be removed.

 *M* = *M*\{mh*}

***Step5 Passenger flow diversion between the bus stops***

***for*** *g* ***in*** *G*

*Divert passenger flow between optimized bus stops so that the bus stop direction is as*

*consistent as possible with passengers’ walking direction heading to it*.

## 4. Case study

### 4.1. Background overview

**Fig 4:**
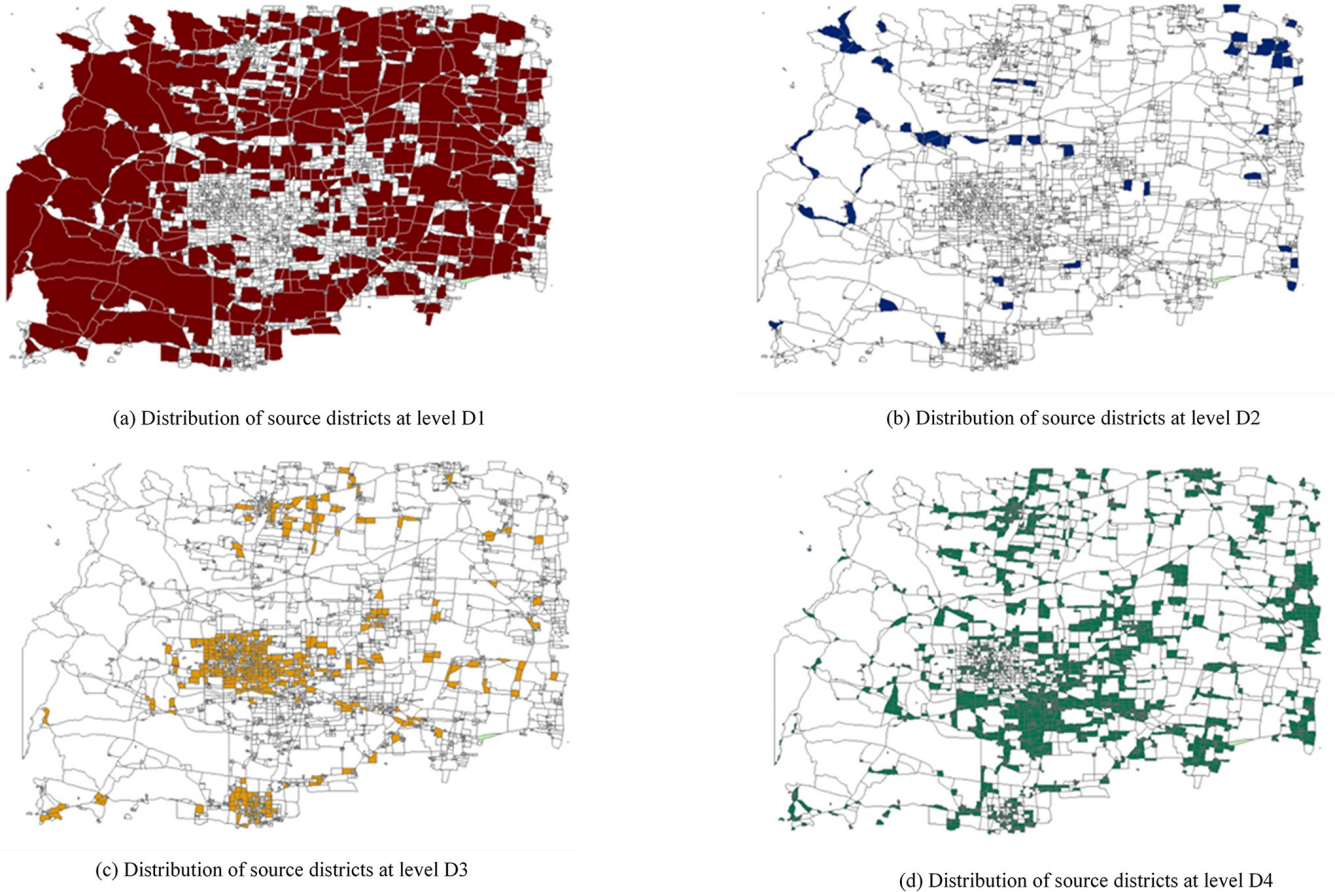
Distribution of source districts by level.

The road network and traffic districts with their population and area data of XT City are input into the GIS system. The study area is divided into 799 traffic districts, as shown in Sheet1 of the S1 Table, and each further divided into several source districts, totaling 5462 source districts. Urban roads are categorized into five levels: expressways, arterial roads, secondary roads, tertiary roads, and other roads. Sorting and statistical analysis of the population and area data of the source districts yield a population threshold of 776, a population threshold coefficient of 0.02220, an area threshold of 2,058,381, and an area threshold coefficient of 0.01780. Based on the population and area scales of the source districts, they are classified into four levels, labeled D1 to D4, as shown in Fig 4. Among them, the D1-level source districts have a large population and a large area, the D2-level source districts have a small population but a large area, the D3-level source districts have a large population but a small area, and the D4-level source districts have both a small population and a small area.

**Fig 5:**
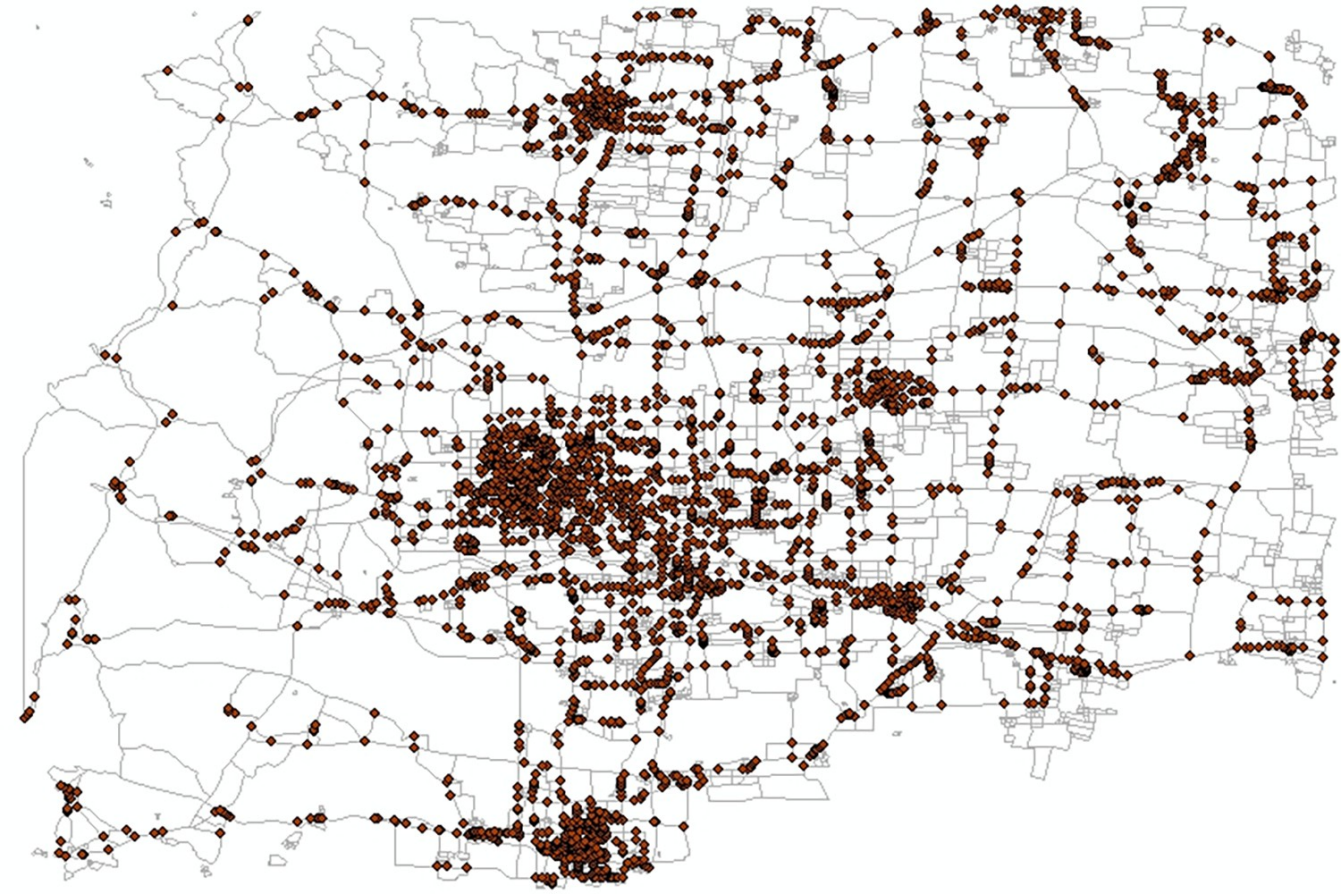
Initial node set.

**Fig 6:**
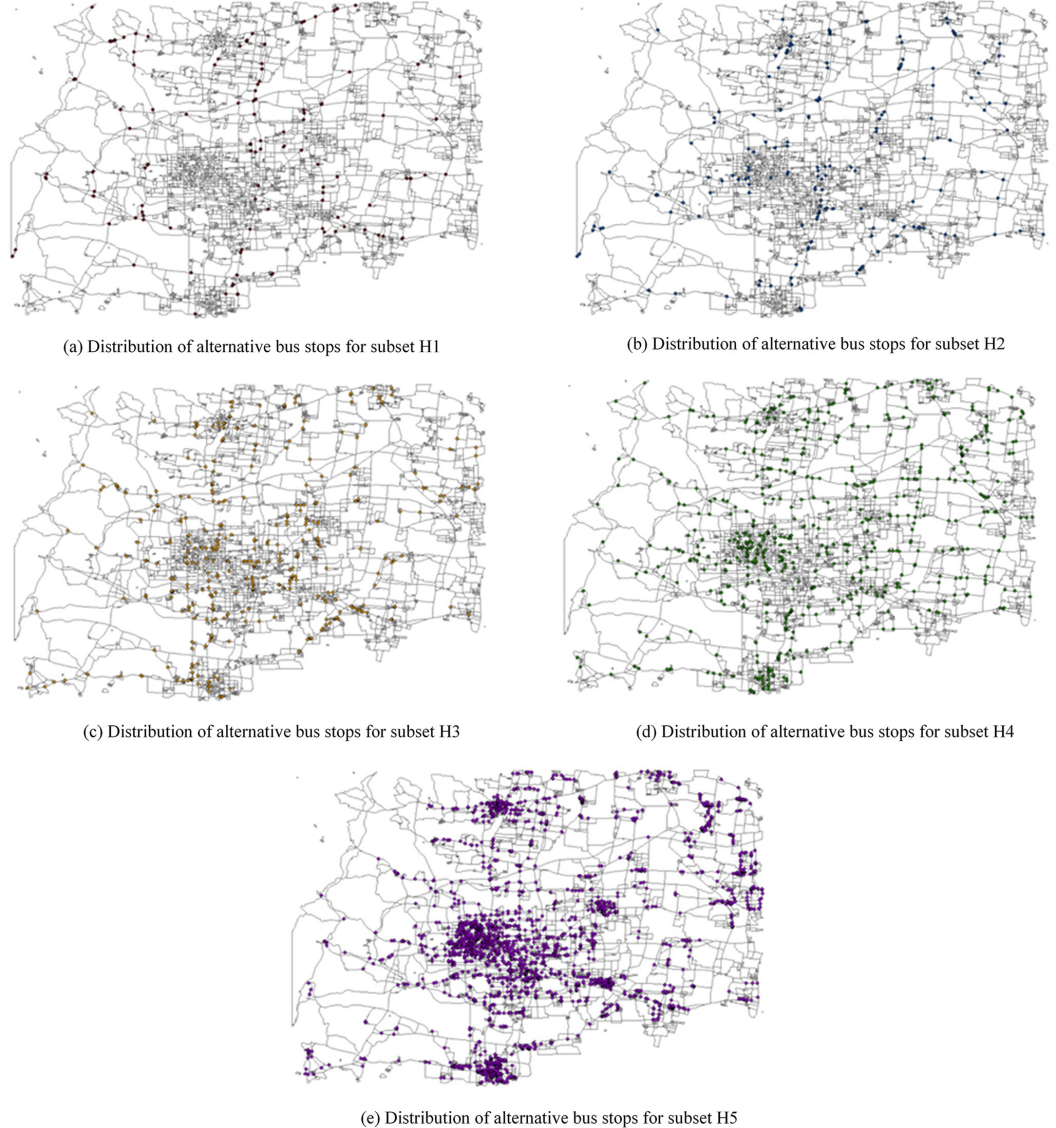
Distribution of hyperedge nodes.

Using the intersections of urban roads and connecting roads, an initial set of nodes is generated, totaling 3,995 nodes, as depicted in Fig 5. Employing the previously mentioned method of node set division based on hypernetwork clustering, combined with the classification of source district types and urban road levels, a hypernetwork is constructed consisting of nodes, source districts, and urban roads. The hypernetwork is then divided into five subsets, representing five hyperedges of the hypernetwork, as illustrated in Fig 6. The weights of each hyperedge in the hypernetwork are calculated to serve as the initial weights for the nodes in the hyperedges, which are 0.53040, 0.21837, 0.19032, 0.16327, and 0.00624, respectively.

**Fig 7:**
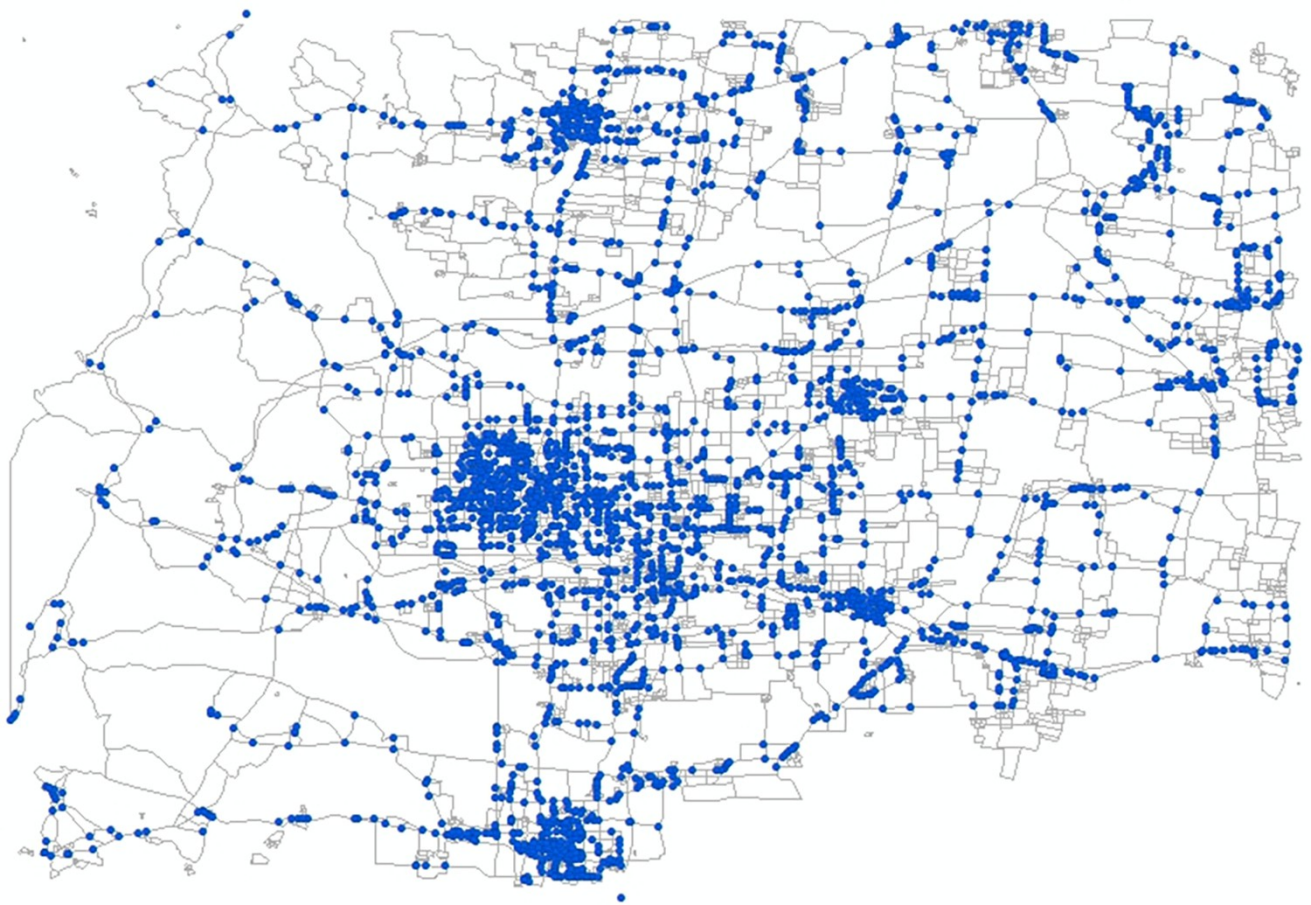
Distribution of alternative bus stops.

With the effective coverage of bus stops as the goal, the nodes of the hypernetwork are merged in the order of hyperedges to generate a final alternative set of bus stops, totaling 2,808 alternative bus stops, as shown in Fig 7.

### 4.2. Case solving

Based on the constructed alternative set of bus stops and passenger flow data, the bus stop layout optimization model is applied to make decisions regarding the building, removal, and moving of bus stops. In our case, the attribution of each stop to the traffic district and the passenger flow between stops are shown in Sheet2 and Sheet3 of the S1 Table. The model is solved using a Python program calling the Gurobi solver, executed with the help of an Intel(R) Core(TM) i3-10100 processor running at 3.60GHZ.

**Fig 8:**
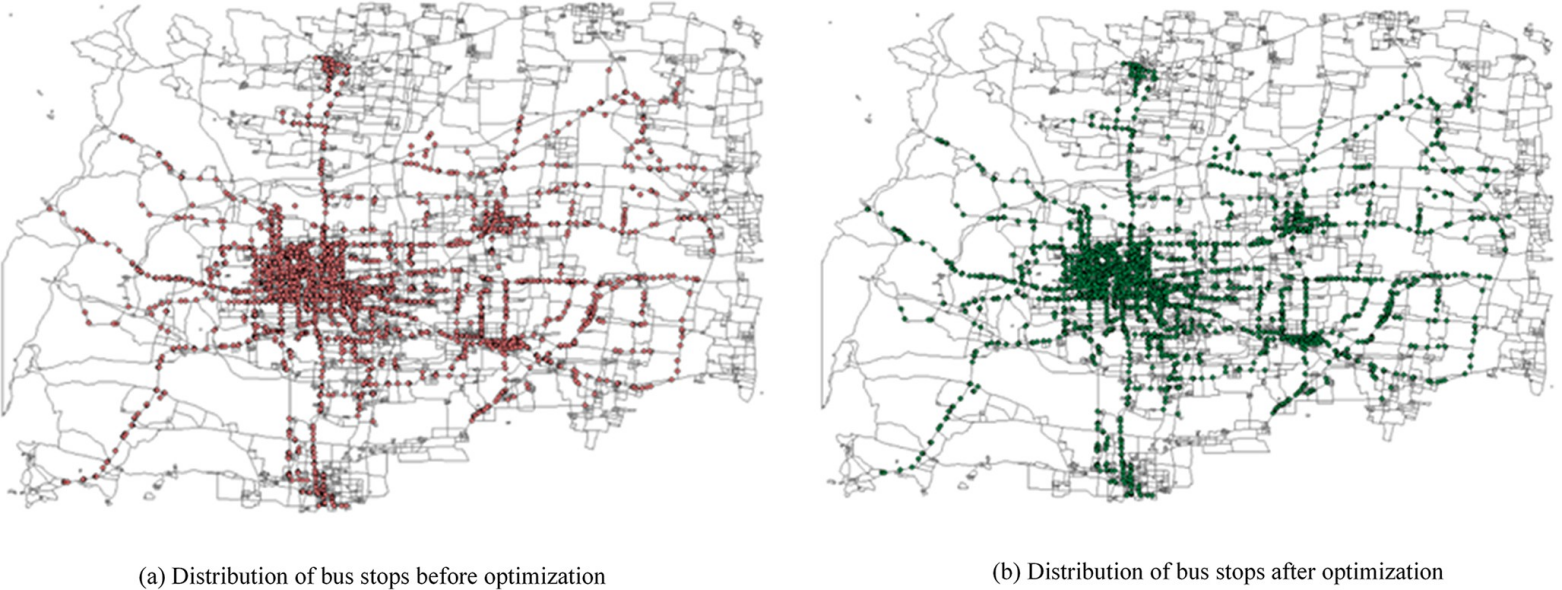
Distribution of bus stops before and after optimization.

The number of bus stops before and after optimization is 2867 and 2874, respectively, as shown in Fig 8.

**Fig 9:**
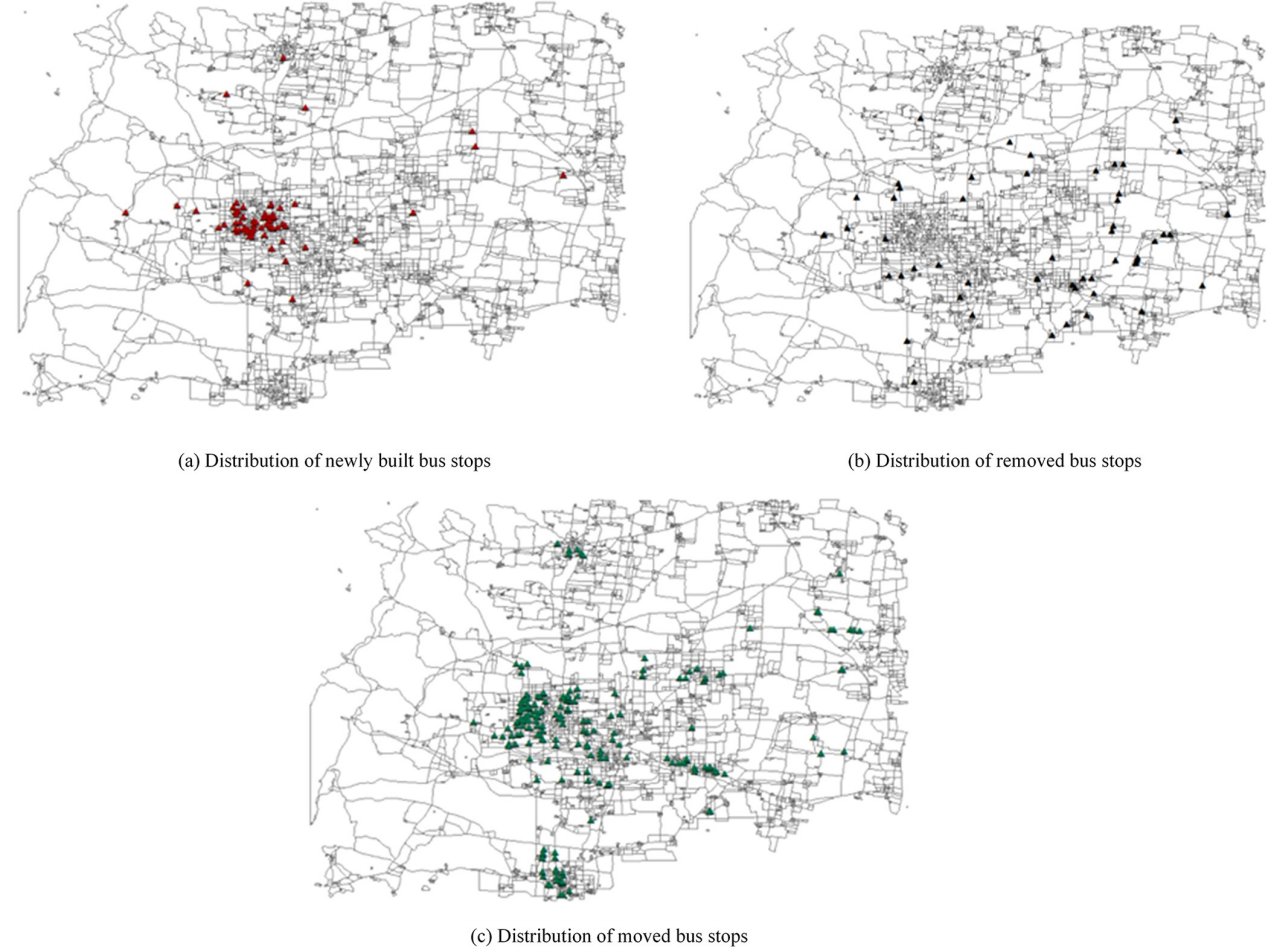
Distribution of newly built, removed, and moved bus stops.

Among these, 64 bus stops are built, 58 are removed, and 189 are moved, with the distribution of stop locations shown in Fig 9.

Preliminary analysis indicates that the newly built stops are primarily located in the central urban area of XT City, where the population is highly concentrated, and passenger travel demand is robust with a complex OD distribution. The building of new bus stops can enhance the matching degree between passenger travel directions and stop settings. The removed bus stops are mainly in the peripheral districts and townships surrounding the central city, where the population is sparse, and passenger travel demand is insufficient. Removing some bus stops can reduce the operating costs of the bus company and improve the travel speed of bus lines. The moved bus stops are mainly in the central city and some of its subordinate districts, where there is a strong demand for passenger travel. Moving unreasonable bus stops can save residents’ travel time.

### 4.3. Indicator analysis

**Fig 10:**
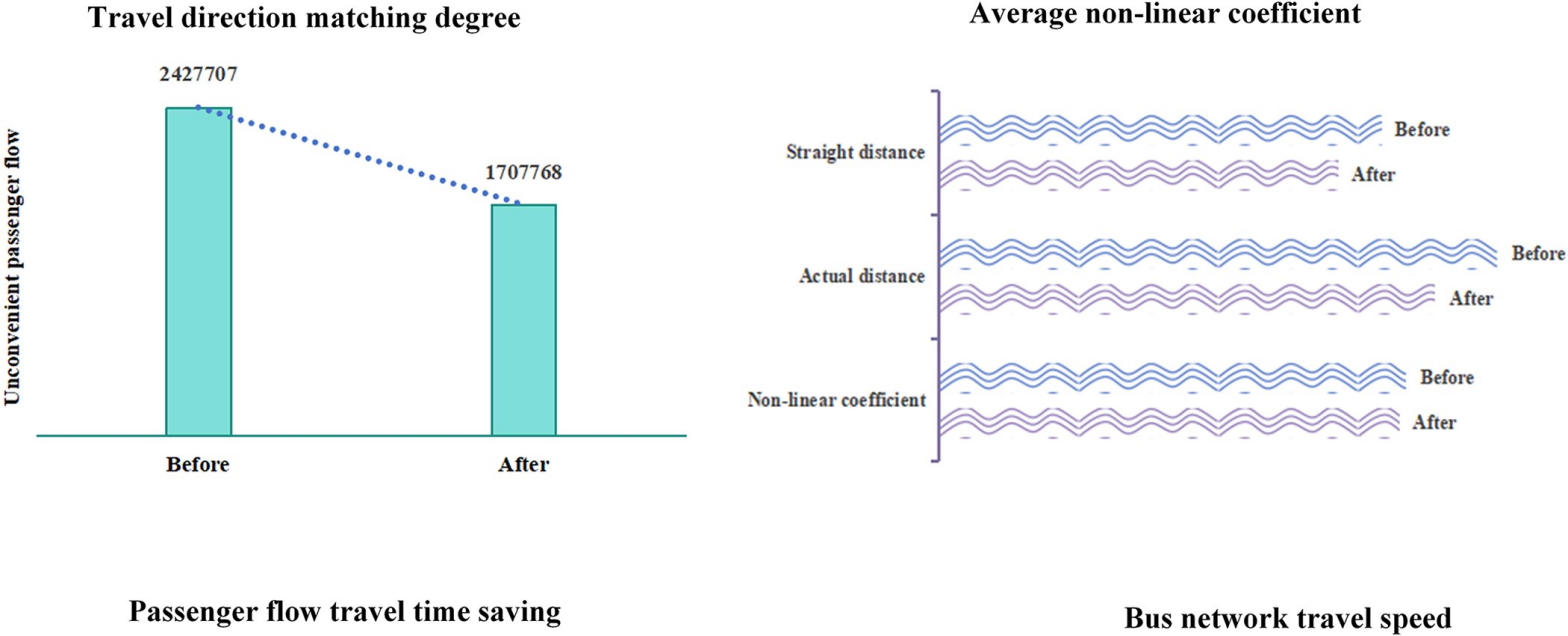
Travel direction matching degree and non-linear coefficient of passenger flow.

**Fig 11:**
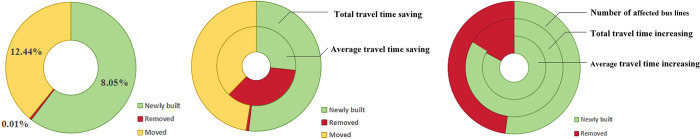
Passenger flow travel time saving and bus network travel speed.

We analyze the results of bus stop layout optimization in XT city from the aspects of travel direction matching degree, average non-linear coefficient, travel time saving of passenger flow, and bus network travel speed. For the matching degree between passenger flow direction and bus stop layout and the average non-linear coefficient of passenger flow, we mainly compare the changes before and after the optimization and form the indicator charts as shown in Table 4 and Fig 10. For the travel time saving of passenger flow and bus network travel speed, we mainly compare the different optimization types and form the indicator charts as shown in Table 5 and Fig 11.

**Table 4:** Travel direction matching degree and non-linear coefficient of passenger flow.

	Travel direction matching degree		Average non-linear coefficient		
	Unconvenient passenger flow	Percentage of total	Non-linear coefficient	Actual distance(m)	Straight distance(m)
**Before**	2427707	53.36%	1.2621	7545.946	5978.958
**After**	1707768	37.53%	1.2439	6709.482	5393.735
**Percentage**	15.83%	15.83%	1.44%	11.08%	9.79%

**Table 5:** Passenger flow travel time saving and bus network travel speed.

	Affected passenger flow		Travel time saving(min)		Affected bus lines	Travel time increasing(s)	
	Number	Percentage	Total	Average	Number	Total	Average
**Newly built**	565871	12.44%	3790521	6.7	59	9660	164
**Removed**	341	0.01%	-3045	-8.93	54	-1860	-34
**Moved**	366116	8.05%	3462892	9.46	-	-	-

1. After the optimization of the bus stop layout, the matching degree between the passenger travel direction and the bus stop layout has been significantly improved. Table 4 shows that the proportion of inconvenient passenger flow before the bus stop layout optimization exceeded 50%, indicating that the bus stop layout in the case does not match the travel direction of citizens very well, and there is a certain degree of detour in the path of passengers; after the optimization, the number of inconvenient passengers decreases from 2427707 to 1707768, accounting for 15.83% of the affected passenger flow. The matching degree between the passenger travel direction and the bus stop layout has been significantly improved, which is conducive to saving passengers’ travel time.
2. The non-straight line coefficient of passenger flow has decreased, indicating more convenient travel for passengers. The non-straight line coefficient for passenger flow is defined as the ratio of the actual travel path distance to the straight-line distance of OD. Table 4 shows that after the optimization, the average non-straight line coefficient of OD has decreased by 1.44%, indicating a reduction in the detour situation of passengers taking the bus. The average actual distance and the average straight-line distance have decreased by 11.08% and 9.79%, respectively, indicating that passengers’ travel time can also be saved.
3. The optimization of the bus stop layout has shortened the travel time of passengers, saving an average of 7.55 minutes of travel time. The travel time of passenger flow within the traffic district is defined as the time from the centroid of the traffic district to walk to the bus stop, and then take the bus along the roads surrounding the traffic district and leave the traffic district. Taking the traffic district numbered 357 as an example, we analyze the impact of optimization operations such as newly building, removing stops, and moving stops on passenger flow, from the aspects of travel time and travel speed.

**Fig 12:**
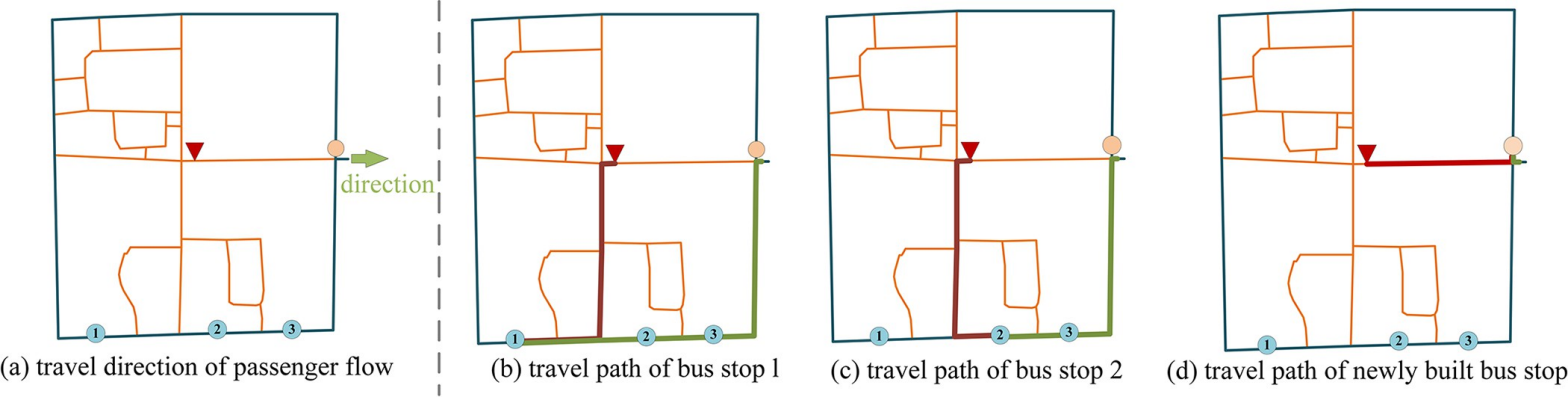
Passenger travel paths of bus stops in District 357.

As shown in Fig 12(A), after the optimization, there are 3 existing bus stops and 1 newly built bus stop in District 357. The blue dots represent the existing bus stops, and the orange dots represent the newly built bus stops. It can be seen from the figure that the existing bus stops in District 357 are mainly located in the south of the district, while the newly built bus stop is located in the east of the district. Therefore, the passenger flow in the east direction will divert from the existing bus stops in the district to the newly built bus stop, and the passenger diversion volume is shown in Table 6.

**Table 6:** Passenger diversion of bus stops in District 357.

From	Into	Passenger diversion volume
Existing bus stop 1	Newly built bus stop	17834
Existing bus stop 2	Newly built bus stop	9897
Existing bus stop 3	Newly built bus stop	0

The travel paths of the above passenger flow from the existing bus stop 1, existing bus stop 2, and the newly built bus stop are shown in Fig 12(B)–12(D), where the red folded line indicates the walking path and the green folded line indicates the path by bus. Taking the pedestrian walking speed as 5km/h and the bus running speed as 15km/h, we can calculate the travel time of the above passenger flow from the existing bus stop 1, existing bus stop 2 and the newly built bus stop as 9.84min, 8.44min and 3.84min respectively, and combined with the amount of passenger transfer volume, we can get the total travel time saved and the average travel time saved as 152530.2min and 5.5min respectively.

From Table 5, it can be seen that the newly built bus stops have the largest impact on the number of affected passengers, and the matching degree between the passenger travel direction and the bus stop setting has been improved, which also brings the largest saving in the total travel time of passengers; in terms of the indicator of average travel time saved, the optimization effect of moved stops is the best, indicating that the moved stops effectively saves the travel time of residents. Analyzing the indicators of removed bus stops, although the average travel time of passengers has increased significantly, it may be due to the fact that most of the removed bus stops are located in the districts and townships, where the traffic districts are relatively large and the residents’ travel time is longer, but the number of affected passengers is very small, accounting for only 0.0075%. Overall, it has not caused an increase in the total travel time of passengers. Overall, after the optimization, the average travel time of passengers has been saved by 7.55 minutes, which can verify the effectiveness of the bus stop layout optimization model.

4. The newly built bus stops have somewhat slowed down the operation speed of the network, but it has reduced the average travel time for passengers. Assuming that bus lines passing through the newly built bus stops must stop at them, and bus lines that originally stopped at the removed bus stops now skip them, the travel time for the bus lines can be calculated. From Table 5, it can be seen that the number of bus lines affected by the newly built and moved bus stops is quite close, but the total travel time and average travel time of the bus network caused by the newly built bus stops are relatively longer compared to the removed bus stops. Combined with the aforementioned analysis of the distribution of optimized bus stops, this is because the newly built stations are mainly located in the central urban area where the bus network is dense, thereby not only saving residents’ travel time but also enhancing the accessibility of the bus network by connecting multiple bus lines.

## 5. Conclusions

This paper proposes a bus stop layout optimization method that considers multiple factors such as passenger flow direction. Firstly, the key factor of the matching degree between passenger flow direction and bus stop layout is innovatively introduced, and the quantification method of this factor is given in the pre-processing of the optimization model, which makes the passenger flow demand more accurate. Secondly, a bus stop alternative set based on hypernetwork is constructed using the hypernetwork multidimensional data clustering method and GIS technology by integrating factors such as traffic district population, area, and urban road conditions. The hypernetwork model constructed based on the network relationship between nodes, source districts, and connecting roads assigns a weight to each alternative stop, and the resulting bus stop alternative set is more scientific and reasonable. Finally, a two-stage optimization model is constructed to improve the matching degree between passenger flow direction and bus stop layout, which can give the optimal adjustment plan for newly building, removing and moving stops by weighing the passenger demand and the optimization cost, and at the same time consider minimizing the negative impact on the travel speed of the bus network. The results of an example in XT city based on the bus stop layout optimization method yielded 64 newly built bus stops, 58 removed bus stops and 189 moved bus stops. The analysis of the indicators shows that the bus stop layout of XT city after the optimization has improved the matching degree of passenger flow direction by 15.83%, the average travel time of the passenger flow has been saved by 7.55min, and the average non-linear coefficient has been decreased by 1.44%. It can be seen that our bus stop layout optimization method can effectively improve the matching degree of passenger flow direction, save passenger flow travel time, and improve passenger flow travel convenience.

Due to data limitations, the passenger flow data that we can obtain is only the information of passengers boarding and alighting from the bus stops, but not the specific information of the most accurate origin or destination of the passengers, thus we adopt an equilibrium strategy, that is calculating the time of passengers between the bus stops and the center of traffic districts. A more reasonable bus stop layout can improve the convenience of passengers’ travel and thus enhance the attractiveness of the bus system, and to a certain extent, it will also generate more new passenger flows, but this paper has not yet investigated the impact on the bus system of the optimized stop layout inducing new passenger flow. The specific settings of bus stops also need to consider micro-level factors, such as their relative position to signal lights or pavements and other traffic facilities, etc. To make detailed adjustments to the bus stops, this paper focuses on the overall optimization of the urban bus stop layout, and does not study the micro-location of the stops. Therefore, in future research, we can obtain more specific passenger flow data, and add the passenger flow induction of bus stops, micro-adjustment, and other aspects of the research, so as to make the optimization of bus stop layout more valuable and meaningful.

## Supporting information

S1 Table Fundamental data of the districts, bus stops, and passenger OD. (XLSX)
